# Metastatic Breast Cancer mHealth App to Promote Patient-Provider Communication: Protocol for a Usability and Satisfaction Study

**DOI:** 10.2196/66050

**Published:** 2025-09-08

**Authors:** Thais F Alves, Kaitlyn Crosby, Ronnie D Horner, Hongying Daisy Dai, Jairam Krishnamurthy, Melanie J Cozad

**Affiliations:** 1 Department of Health Services Research & Administration College of Public Health University of Nebraska Medical Center Omaha, NE United States; 2 College of Public Health University of Nebraska Medical Center Omaha, NE United States; 3 Department of Internal Medicine College of Medicine University of Nebraska Medical Center Omaha, NE United States

**Keywords:** mHealth, breast cancer, patient-provider communication, usability study, mixed methods research, metastatic, mHealth app, patient-provider, communication, usability, protocol, mobile health, digital, technology, patient outcome, satisfaction, cross-sectional, semi-structured interview, design, modification

## Abstract

**Background:**

With the availability of more advanced and effective treatments, life expectancy has improved among patients with metastatic breast cancer (MBC), but this makes communication with their medical oncologist more complex. Some patients struggle to learn about their therapeutic options and to understand and articulate their preferences. Mobile health (mHealth) apps can enhance patient-provider communication, playing a crucial role in the diagnosis, treatment, quality of life, and outcomes for patients living with MBC. Our team developed an app called My MBC Journey to focus on the collection of important information for patients with MBC between clinical encounters.

**Objective:**

This study will evaluate the usability and satisfaction of My MBC Journey, a mobile app designed for MBC, to inform future modifications.

**Methods:**

This mixed methods study will assess patient use and satisfaction with the My MBC Journey app to inform future app modifications and identify the barriers and facilitators to the app’s use for enhancing patient-provider communication. We will recruit a prospective, cross-sectional convenience sample of 25 patients with MBC and a sample of 15 members of the care team (ie, caregivers, nurse navigators, and medical oncologists). Applying iterative, convergent mixed methods, we will conduct qualitative, semistructured interviews with the patients and care team members. We also will collect quantitative data on usability through app analytics and standardized questionnaires (ie, the Mobile Application Rating Scale, the Mobile Application Rating Scale user version, and the System Usability Scale). Qualitative interviews will be audio recorded and analyzed using NVivo software to identify mHealth implementation themes.

**Results:**

The study’s results will inform future app design modifications and gauge preliminary effect size in support of future evaluations of the app’s efficacy in improving patient-provider communication.

**Conclusions:**

Our long-term goal is to improve patient-provider communication by developing mHealth apps that empower patients to collect and share clinically relevant, patient-reported information in a timely manner.

**International Registered Report Identifier (IRRID):**

PRR1-10.2196/66050

## Introduction

Metastatic breast cancer (MBC) is late-stage breast cancer, where the disease has spread beyond the nearby lymph nodes to other sites such as the liver, lungs, bones, and brain [1,2]. Although men develop breast cancer, MBC most often affects women [3]. It is estimated that 168,000 women in the United States are living with MBC, and that number will likely grow to around 246,000 by 2030 [4,5]. Previously, treatment of MBC was nihilistic in nature, with a major goal of care being palliative. However, with the availability of more advanced and effective treatments, life expectancy has improved among patients with MBC, with over one third of patients now living 5 years beyond their diagnosis and some exceeding 15 years of survival [6,7].

Given the many therapeutic options now available, prolonging survival is a feasible goal for many individuals with MBC; however, it also makes communication with their medical oncologist more complex and essential to the treatment process [7]. Many patients struggle to identify and articulate their preferences for therapeutic options that may help them achieve their personal goals for living with cancer. Current guidelines from the National Comprehensive Care Network involve over 45 treatment regimens for MBC that include chemotherapy (single-agent or combination), hormonal therapy, and immunotherapy [8]. For patients receiving treatment beyond the recommended first-line therapies, the therapeutic combinations present many different toxicity considerations [9]. Beyond the number of potential therapeutic options, communication and decision-making between patients and their medical oncologists is increasingly complex due to competing medical comorbidities and acceptability of treatment toxicities. Patients want to participate in treatment conversations with their medical oncologists, but they often feel overwhelmed by the magnitude of treatment information and how it aligns with their personal goals [6,7]. Additionally, they may undervalue their own insights and are hesitant to ask questions about problematic side effects [6,7]. Patients also must balance the demands of their therapeutic program and its consequences with their health-related social needs, such as housing, food, transportation difficulties, and personal well-being [6,7,10].

Mobile health (mHealth) apps represent a potential tool to facilitate communication between the patient and the medical oncologist. These apps allow the patient to identify, record, and recall information about their well-being, personal goals, and toxicity preferences between clinical visits on their smartphone [11]. Given the high percentage of individuals with a mobile phone that they carry everywhere, the app could assist with recalling and sharing this information during the clinical encounter. Mobile apps also may reach a broader, more diverse patient population, including those who have historically experienced disparities in patient-centered care [11,12]. However, evidence on digital health interventions (DHIs) to improve patient provider communication is limited. A recent systematic review identified only 13 DHIs that targeted improvement of patient-provider communication in cancer care, with just 4 focused on breast cancer. None of these interventions involve delivery through a mHealth app [13]. Additionally, in 2023 we conducted a preliminary review of existing mHealth apps for breast cancer and identified only 1 for MBC that was available in the United States. Upon initial review, this app did not include functionality for a DHI focused on enhancing communication with the provider. In a subsequent review of this app in the fall of 2024, it was no longer available within the app store. With no apps available for patients with MBC in the United States, there is a clear need for continued, evidence-based development of mHealth apps for patients with MBC.  

Our mHealth app, My MBC Journey, was designed to focus on the collection of important information to the patient with MBC between clinical encounters. Collection and organization of information important to the patient may help enhance self-efficacy and build confidence towards communication with the medical oncologist. To design an mHealth app for MBC, our team integrated evidence from qualitative interviews with a human change behavior theory to determine functionality that promotes a patient’s understanding and communication about their health status. The development of My MBC Journey was based on social cognitive theory constructs of self-efficacy and self-regulation [14]. Besides providing the rationale behind the content and functions development process, it is possible to see social cognitive constructs associated with the design of specific resources, such as motivational and personalized messaging and alerts to help people manage their conditions [15,16]. Our app includes the following functionalities: 1) tracking physical, social, emotional, and functional well-being through patient reported outcomes (using the Functional Assessment of Cancer Therapy-Breast and European Organization for the Research and Treatment of Cancer Quality of Life Questionnaire-Breast Cancer 23 instruments); 2) recording personal goals for activities of daily living and treatment preferences for symptom and side effect management; and 3) documenting details about problematic symptoms or side effects in a written, free-form manner. The app also can graphically display patient-reported outcomes (PROs) over the multiple dates where the patient has entered information, thereby demonstrating trends in improvement or decline. It also displays written free-form notes recorded over the same dates as the PROs on top of the graphical display of trends. This novel integration of trends in PROs displayed with the users’ own comments on their health status may help the patient pinpoint how they are feeling at a specific time, thereby enabling more detailed recall and discussion of this information during the clinical encounter. Additionally, having the ability to log goals for activities of daily living and treatment preferences supports the patient in having this information readily available and may enhance their ability to discuss it during the clinical encounter. The app development process, including description of frameworks, screenshots, and functions, is described in depth in a forthcoming paper.

Effective use of mHealth apps as DHIs to improve patient-provider communication hinges on the end users’ engagement with the app and willingness to enter information at a frequency that is clinically valuable. Therefore, as an initial step to app development, this study will evaluate usability and satisfaction with the mHealth app and its barriers and facilitators toward enhancing patient-provider communication. Our specific aims are to assess patient use and satisfaction with the My MBC Journey app to inform future app modifications and to identify the barriers and facilitators to the app’s use for enhancing patient-provider communication.

## Methods

### Study Design

**Figure 1:**
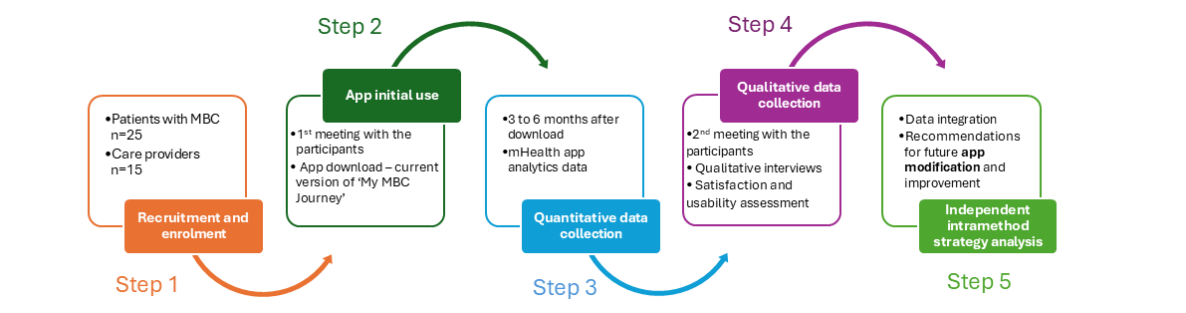
Study design. MBC: metastatic breast cancer; mHealth: mobile health.

This study will use a prospective, convergent mixed methods design to assess the usability and satisfaction of an mHealth app for patients with MBC that was developed to promote patient-provider communication [17]. We will collect and integrate quantitative and qualitative outcomes on app use and satisfaction with functionality (aim 1). For Aim 2 we will use qualitative, personal interviews to identify barriers and facilitators of the app’s use to determine how it may best be integrated into the clinical care setting to enhance patient-provider communication [18]. We will seek both the patients’ and the care team members’ perceptions. The results will inform modifications to our mobile app that will then be ready for future, iterative usability testing and eventual evaluation of the app’s efficacy to enhance patient-provider communication (Figure 1).

### Participants and Recruitment

Patients with MBC and their care team members are eligible to participate in this study. Aim 1 will require a prospective, cross-sectional convenience sample of 25 participants with MBC. Aim 1’s recruitment inclusion criteria are as follows: patients aged 19 years or older who are able to speak and read English, have clinically-documented MBC, possess a mobile device on which the mHealth app for MBC can be installed (Android or iOS operational systems), have a visit with their medical oncologist within the next 4 months, and are able to provide written informed consent. Exclusion criteria are participants who decide with their oncologist to enter hospice care. Similar criteria have been used in previous studies determining mobile apps’ usability by patients with other diseases [19].

Aim 2 requires a prospective, cross-sectional convenience sample of care team members who provide oncologic care to the study participants recruited in aim 1. A sample of 15 care team members will be recruited. A breast cancer care team may consist of medical oncologists, nurse navigators, informal caregivers, radiation oncologists, psychiatrists, physiologists, nutritionists, social workers, and integrative therapists, among others [20]. In this study, eligible care members include medical oncologists, nurse navigators, and informal, patient caregivers because they are most likely to assist and engage with the patient during usage of the app, and hence, will experience how the apps’ functionality potentially enhances or inhibits patient-provider communication at the point of care.

Our team will partner with the Buffet Cancer Center in Omaha, Nebraska, to register eligible participants over a 7-month period and follow them for at least 1 visit with their medical oncologist. Frequency of visits for patients with MBC is heterogenous, based upon disease severity such as whether it is in an acute or stable phase. Most patients within our setting see their medical oncologist every 3 to 6 months [19]. While this time window may cause different exposure length to the app, it is necessary to support collection of data regarding patients’ perceptions about how the app functions to enhance patient-provider communication in aim 2. Potential patient participants will be informed of the study by their medical oncologist during a regular visit. The medical oncologist and research team will work to ensure diversity across clinical and demographic strata and mitigate sample bias to the extent possible. If the patient is willing to consider participation in the study, the oncologist will give the patient a flyer with written information that explains the study and provides contact information for further details. The contact, referred to hereafter as the study coordinator, will field inquiries (by phone call or text). For the participant who indicates a willingness to participate, the study coordinator will review the aspects of the study and obtain informed consent from the participant during the phone call or a web-based meeting. Once the participant provides informed consent by reviewing and electronically signing the consent form on Research Electronic Data Capture (REDCap) [21], the study coordinator will schedule a meeting to assist the participant in downloading and using the mobile app. 

Care team members also will be recruited from within the Buffet Cancer Center. Medical oncologists and nurse navigators will be informed about the study through flyers posted within the cancer center and snowball recruitment. Informal caregivers will be recruited via nomination of a patient participant in the study, snowball recruitment, or through posted flyers of the study. Care team members may be part of a care team dyad but do not have to be part of that dyad to participate. For care team members who indicate a willingness to participate, the study coordinator will review the aspects of the study and obtain informed consent from the participant during the phone call or a web-based meeting. Once the care team member participant provides informed consent by reviewing and electronically signing the consent form on REDCap, the study coordinator will schedule a meeting to familiarize the care team member with the app and conduct a qualitative interview. Familiarization with the app will occur through demonstrating the major features of the app in a web-based testing environment. Additionally, care team members will be given time to navigate through the app at their own pace and ask questions about how the app works for the patient. During the same session, a study team member will conduct a qualitative interview.

### Study Procedure (Data Collection of Outcomes)

Data collection of outcomes will occur in 2 ways: via the app and through in-person interviews (Table 1). Outcomes collected via the app include aggregated data analytics, such as how many times, on average, users went to a page and how long they spent on the page (aim 1a). During the in-person interviews, we will collect the qualitative outcomes and use the following standardized questionnaires to assess satisfaction and usability (aims 1b, 1c, 2a, 2b, and 2c): the Mobile Application Rating Scale (MARS) [22] with care team participants, the Mobile Application Rating Scale user version (uMARS) [23] with patient participants, and the System Usability Scale (SUS) [24,25] with both patients and care team members. The interviewer will provide an explanation about the standardized questionnaires used to measure satisfaction and usability, and they will be self-administered by the participants. Interview guide questions will be developed based on a matching integration strategy applied from a convergent mixed method research methodology [17] to gather more in-depth data about app use. 

During the initial use of the app, the participant will provide the independent variables (questions about age, sex, race and ethnicity, education, marital status, income, health-related social needs, and whether they have ever used another health app); the study coordinator will be available to assist the participant as required. As participants use the app, we will obtain outcome measures for aim 1a. After the app use period is completed and the patients with MBC have a visit with the medical oncologist (approximately 4 months), our team will contact the participants (patient and care team member) to schedule the interviews and collect outcomes for aims 1b, 1c, 2a, 2b, and 2c, including, for example, completion of the standardized MARS, uMARS, and SUS questionnaires [22-25]. Interviews will be scheduled for 1 to 2 weeks following the patient’s appointment with the oncologist depending on their availability. The care team members outcomes and independent variables (age, sex, and race and ethnicity) will also be collected during the interviews. Each interview will take up to 60 minutes, and with participant consent, will be audio recorded. The interviews will be conducted by a researcher with a clinical background and extensive experience in qualitative data collection and digital health evaluation. To avoid personal bias, the team member responsible for qualitative data collection will have no previous knowledge, contact, or work experience with the patients or care team members in any capacity.

The audio records will be transcribed using the support of NVivo software (Lumivero) for further analysis. Participants will receive a $30 gift card for participation. 

**Table 1:** Outcome measures.

Outcome		Source of data	Measures	When the outcome is collected
**Aim 1: access patient use of the mHealth^a^ app to inform future modifications using iterative, convergent mixed methods.**				
	1a. Identify the app functions used most often and to what extent	Quantitative data on mHealth app analytics	Events (number of times a page within the app was visited, amount of time participants remained there, and average views on a page per user) and duration (amount of time a patient-reported measure took to complete; assessed using the FACT-B^b^ or EORTC QLQ-BR23^c^)	As participants use the app after initial download.
	1b. Assess patient perceptions of the app’s aesthetics and content and their sustained engagement with the app	Qualitative personal interviews and quantitative survey questionnaires	Open-ended question prompts (10 items) and uMARS^d^ (20 items)	Within 3 to 6 months after app download during a semistructured personal interview after a visit to a medical oncologist.
	1c. Assess patients’ satisfaction with the mobile app functionality	Qualitative personal interviews and quantitative survey questionnaires	Open-ended question prompts (10 items) and SUS^e^ (10 items)	Within 3 to 6 months after app download during a semistructured personal interview after a visit to a medical oncologist.
**Aim 2: assess the barriers and facilitators to app use for enhancing patient-provider communication.**				
	2a. Assess patient perceptions of app use to promote communication with their medical oncologist	Qualitative personal interviews	Open-ended question prompts (8 items)	Within 3 to 6 months after app download during a semistructured personal interview after a visit to a medical oncologist.
	2b. Assess the care team members’ perceptions of the app and ways in which it is useful to them in supporting the patient	Qualitative personal interviews	Open-ended question prompts (8 items)	Following interactions with the patients and app after download. Within 3 to 6 months after app download.
	2c. Assess the care team members’ satisfaction with functionality, their perceptions of the app’s aesthetics and content, and their sustained engagement with the app	Quantitative survey questionnaires	MARS^f^ (23 items) and SUS (10 items)	Within 3 to 6 months after app download.

^a^mHealth: mobile health.

^b^FACT-B: Functional Assessment of Cancer Therapy-Breast.

^c^EORTC QLQ-BR23: European Organization for the Research and Treatment of Cancer Quality of Life Questionnaire-Breast Cancer 23.

^d^uMARS: Mobile Application Rating Scale user version.

^e^SUS: System Usability Scale.

^f^MARS: Mobile Application Rating Scale.

### Data Analysis

To analyze the outcomes related to the extent of the app’s use (aim 1a) and the standardized questionnaires (aims 1b, 1c, and 2c), we will use descriptive statistics such as means, SDs, and tabulations. Specifically for the usability and satisfaction classification and rating, we will follow the recommendations for scoring of each instrument [22,23,25]. For the qualitative outcomes explored through the interviews, we will analyze the transcripts by applying content analysis [26] in 4 stages—decontextualization, recontextualization, categorization, and compilation—using the software NVivo. To avoid personal bias and to increase validity, after reviewing 2 transcripts, a codebook will be established independently by 2 researchers with expertise in qualitative analysis who will meet to garner consensus across the codebook and then apply it to a third transcript and discuss any further changes necessary. Upon project leader concordance with the codebook, the 2 investigators will analyze the remaining transcripts separately and then discuss their results to obtain consensus.  

Our research team will apply the independent intramethod strategy from the convergent mixed methods design to analyze the outcomes of this mixed method study. With this strategy, the qualitative data analysis occurs independently of the quantitative data analysis, and then the findings from both are integrated to draw overarching interpretations and formulate recommendations for future mobile app modification [17] (Figure 1). Qualitative and quantitative data will be examined within a joint-display table to compare the similarities, differences, or contradictions [17]. Convergence of data between methods highlights areas for app modification or demonstrates that the app’s development is satisfactory and usability testing is complete. In cases of discordance, both qualitative and quantitative data will be reanalyzed to resolve differences based on the social cognitive theory constructs used during app development and the usability and satisfaction objective quality items addressed in the MARS, uMARS, and SUS questionnaires. 

### Sample Size Consideration

Our feasibility test will apply the previously piloted methods to recruit 25 patients with MBC and a sample of 15 members of the care team (ie, caregivers, nurse navigators, and medical oncologists). This sample size is based on guidelines for behavioral treatment development research [27]. Our goal is to get reasonable estimates of the intervention's effect size for planning the needed sample size in future usability testing. Thus, we performed a power analysis in PASS 14 (NCSS Statistical Software) to calculate the CIs of the utility measure estimates.

**Figure 2:**
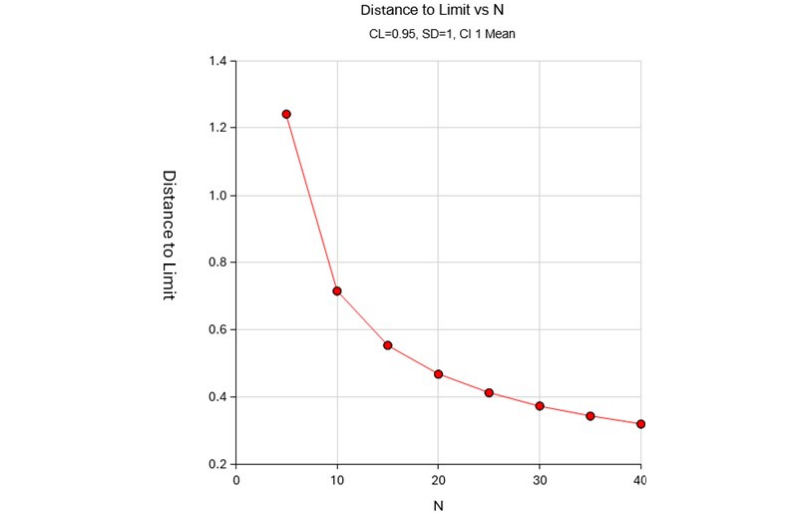
Sample size determination based on the CI of the mean. CL: confidence level.

The confidence level represents the proportion of CIs that would contain the population mean when constructed with the same parameters. A CI effectively measures estimation precision. Figure 2 shows the distance to the limit for sample sizes ranging from 5 to 40, demonstrating that increasing sample size enhances estimation. Distance from mean to limit refers to the distance between the confidence limits and the mean. For 2-sided intervals, this is also called precision, half-width, or margin of error. The target distance is the specified value entered into the procedure, while the actual distance is the value obtained. The population's SD measures its variability.

We calculated the sample size using the CI formula for 1 mean and generated numeric results for 2-sided CIs with an unknown SD. A sample size of 25 produces a 2-sided 95% CI with a distance from the mean to the limits of 0.41 when the estimated SD is 1 [28]. This sample size ensures that the actual distance to the limit is below the target distance, set at 0.5. The calculation also accounts for a 20% dropout rate. Figure 2 shows the distance to the limit for sample sizes ranging from 5 to 40, demonstrating that increasing the sample size enhances estimation.

For the care team members’ sample, we selected to obtain a sample size of 15. This sample size should be sufficient to reach saturation of themes for barriers and facilitators of the app’s functionality to inform app modifications for future useability testing for the care team as a whole. Future research will focus on cross comparisons between medical oncologists, nurse navigators, and informal caregivers.

### Data Management Plan

The data elements recorded through the mobile app will be stored in a secure, cloud-based server. The mobile app passes the data—identified only by the unique numeric identifier assigned to each participant—to a secure, cloud-based storage system that is accessible only by the study leads. All information in the secure cloud storage system is deidentified, as this study requires no names or personal identifiers of the participants to be collected. All interviews and other related data (such as the researcher’s note) from the qualitative, semistructured interviews will be stored under the unique numeric participant identifier, allowing app-derived data and interview-derived data to be linkable. If the participant wishes to be compensated with the gift card, we will collect information on name and address solely for this purpose, and this list will not be linked to any of the data collected from the participant. We will keep the data for 7 years in a secure shared folder accessible only to the study leads. 

### Ethical Considerations

The mobile app, My MBC Journey, was approved by the University of Nebraska Medical Center Information Technology security team for use. Our study is under review by the University of Nebraska Medical Center Institutional Review Board (#0791-23-EP). We will initiate the data collection process only after receiving IRB approval. We received financial support from the National Cancer Institute through an administrative supplement focused on digital health and patient-provider communication through a P30 grant awarded to Dr. Kenneth Cowan at the Fred and Pamela Buffet Cancer Center. 

## Results

We anticipate the enrolment of 40 participants (25 patients with MBC and 15 of their care team members). We expect to enroll the first participants by July 2026 and have 100% of planned enrolment recruited by July 2027. The target completion date of our primary endpoint data analysis is August 2027 and the results will be reported at ClinicalTrials.gov by March 3, 2028. 

## Discussion

### Study Overview

Assessing the usability and satisfaction of the mHealth app, My MBC Journey, will allow us to identify future design modifications and assess the technology’s efficacy through a randomized controlled trial. Our team’s long-term goal is to improve patient-provider communication by developing mHealth apps that empower patients to collect and share clinically relevant, patient-reported information during the clinical visit. The iterative, convergent mixed methods design will provide us qualitative and quantitative data that, when integrated with outcomes from aims 1 and 2, will give us in-depth information on the perspectives of the patients and their care team members, which is essential to achieve better patient-provider communication.

This study is relevant because mobile app use may provide additional clinically relevant information related to patient self-monitoring and satisfaction, thereby activating them in their care and supporting more effective patient-provider communication. We anticipate that participants may also derive satisfaction from knowing their experience could help future patients and health care providers enhance communication. They may also derive satisfaction from helping create a mobile app to improve the quality of life and care for patients with MBC.

### Methodological Limitations

This study will assess usability and satisfaction to track and design future modifications to the mHealth app, but we will not test the app’s effectiveness at this point. Also, this version of the app is only for English speakers and those who can read, but we will seek diversity in the sample across important socioeconomic dimensions (eg, rural- and urban-residing participants). We understand that recruitment and retention of patients with this condition can be a challenge, and we will partner with oncologists to help enroll participants. 

### Methodological Strengths

We will conduct robust mixed methods research, applying the independent intramethod strategy [14] that is specifically designed and validated to evaluate the usability of mHealth apps. Standardized and internationally disseminated questionnaires help ensure the validity of the results, especially for the study’s quantitative portion. Furthermore, our research team has expertise in qualitative analysis, which will allow us to approach the phenomenon and data integration in depth. The strong partnership between the investigators and the cancer center that is the study's setting is also a strength in that it can promote recruitment, enrollment, and participant retention. The results will allow us to improve our technology for use in an assessment of the app’s efficacy in improving patient-provider communication through a randomized controlled trial as a next step. 

## Abbreviations

DHI: digital health intervention
MARS: Mobile Application Rating Scale
MBC: metastatic breast cancer
mHealth: mobile health
PRO: patient-reported outcome
REDCap: Research Electronic Data Capture
SUS: System Usability Scale
uMARS: Mobile Application Rating Scale user version
